# Mechanism of the Potential Therapeutic Candidate *Bacillus subtilis* BSXE-1601 Against Shrimp Pathogenic Vibrios and Multifunctional Metabolites Biosynthetic Capability of the Strain as Predicted by Genome Analysis

**DOI:** 10.3389/fmicb.2020.581802

**Published:** 2020-10-26

**Authors:** Dongdong Wang, Jiahui Li, Guoliang Zhu, Kun Zhao, Wenwen Jiang, Haidong Li, Wenjun Wang, Vikash Kumar, Shuanglin Dong, Weiming Zhu, Xiangli Tian

**Affiliations:** ^1^The Key Laboratory of Mariculture, Ocean University of China, Ministry of Education, Qingdao, China; ^2^Lab of Aquaculture & Artemia Reference Center, Department of Animal Sciences and Aquatic Ecology, Faculty of Bioscience Engineering, Ghent University, Ghent, Belgium; ^3^State Key Laboratory of Bioreactor Engineering, East China University of Science and Technology, Shanghai, China; ^4^Key Laboratory of Marine Drugs, Ministry of Education of China, School of Medicine and Pharmacy, Ocean University of China, Qingdao, China; ^5^Laboratory for Marine Drugs and Bioproducts of Qingdao National Laboratory for Marine Science and Technology, Qingdao, China

**Keywords:** amicoumacin A, genome sequence, *Bacillus subtilis*, AHPND, vibriosis, *Litopenaeus vannamei*

## Abstract

The global shrimp industry has suffered bacterial diseases caused mainly by *Vibrio* species. The typical vibriosis, acute hepatopancreatic necrosis disease (AHPND), has resulted in mass mortality and devastating economic losses. Thus, therapeutic strategies are highly needed to decrease the risk of vibriosis outbreaks. Herein, we initially identified that the growth of the causative agent of AHPND, *Vibrio parahaemolyticus* (VP_*AHPND*_) and other vibrios in Pacific white shrimp (*Litopenaeus vannamei*) was inhibited by a *Bacillus subtilis* strain BSXE-1601. The natural products amicoumacins A, B, and C were purified from the cell-free supernatant from the strain BSXE-1601, but only amicoumacin A was demonstrated to be responsible for this anti-*Vibrio* activity. Our discovery provided the first evidence that amicoumacin A was highly active against shrimp pathogens, including the representative strain VP_*AHPND*_. Furthermore, we elucidated the amicoumacin A biosynthetic gene cluster by whole genome sequencing of the *B. subtilis* strain BSXE-1601. In addition to amicoumacin A, the strain BSXE-1601 genome harbored other genes encoding bacillibactin, fengycin, surfactin, bacilysin, and subtilosin A, all of which have previously reported antagonistic activities against pathogenic strains. The whole-genome analysis provided unequivocal evidence in support of the huge potential of the strain BSXE-1601 to produce diverse biologically antagonistic natural products, which may facilitate further studies on the effective therapeutics for detrimental diseases in shrimp.

## Introduction

The greatest challenge in the global shrimp industry is a disease particularly that of bacterial origin and chief among these is *Vibrio* species (Ajadi et al., 2016; Hoseinifar et al., 2018). The acute hepatopancreatic necrosis disease (AHPND), formerly known as shrimp “early mortality syndrome” (EMS), is a newly emerging vibriosis that has caused severe mortality (up to 100%) and devastating economic effect in the global shrimp industry (Lightner et al., 2012; Joshi et al., 2014; Kongrueng et al., 2015; Boonsri et al., 2017). Infected shrimp show slow growth, an empty stomach and midgut, and severe atrophy of hepatopancreas (Joshi et al., 2014; Kongrueng et al., 2015; Sirikharin et al., 2015; Han et al., 2020). Originally, the etiological agent of AHPND has been reported to be *Vibrio parahaemolyticus* (VP_*AHPND*_) (Tran et al., 2013). Recent studies have shown that other *Vibrio* spp., such as *Vibrio punensis* (Restrepo et al., 2018), *Vibrio owensii* (Liu et al., 2015), *Vibrio harveyi*-like (Kondo et al., 2015), and *Vibrio campbellii* (Dong et al., 2017a,b) are also capable of inducing AHPND in shrimp. Besides AHPND, other vibrioses were frequently reported in farmed shrimp caused by *Vibrio alginolyticus*, *Vibrio anguillarum*, *V. harveyi*, *Vibrio vulnificus*, *Vibrio splendidus*, *V. campbellii* and *Vibrio fischeri* (Lavilla-Pitogo et al., 1990; Lightner, 1996; Lavilla-Pitogo et al., 1998; Chen et al., 2000; Jayasree et al., 2006; Longyant et al., 2008; Zheng et al., 2016; Chandrakala and Priya, 2017; Karnjana et al., 2019). Furthermore, non-*Vibrio* species such as *Aeromonas* spp. (Dierckens et al., 1998; Zhou et al., 2019), *Streptococcosis* spp. (Hasson et al., 2009), *Shewanella* spp. (Wang et al., 2000), *Flavobacterium* spp. (Chandrakala and Priya, 2017) and *Pseudoalteromonas* spp. (Zheng et al., 2016) were also documented to cause severe diseases in fish and shrimp aquaculture. Hence, strategies that focus on restraining the growth or activity of pathogenic bacteria are highly needed.

Recently, *Bacillus* spp. have been suggested to present a high interest as a promising therapeutic agent against vibriosis and other diseases in aquaculture, because of their antagonistic properties against aquatic pathogenic microorganisms, and their non-pathogenic and non-toxic characteristics (Rengpipat et al., 2003; Aly et al., 2008; Ran et al., 2012; Hoseinifar et al., 2018; Kuebutornye et al., 2019; Wang et al., 2019). The bacteriostatic properties of *Bacillus* spp. are linked primarily to the bioactive natural products produced by themselves (Kuebutornye et al., 2019). These natural products are not necessary for the survival of their producers but offer advantages under particular environmental conditions (Hug et al., 2020). They are highly optimized in the course of evolution for interactions with biological targets and, thus, exhibit various biological functions, such as antibacterial, antifungal and immunosuppressive activities (Hug et al., 2020). Genus *Bacillus* can produce a large variety of biologically active natural products, including non-ribosomal peptides (NRPs) (e.g., bacillibactin, bacilysin, fengycin, surfactin), polyketides (PKs) (e.g., bacillaene, difficidin, and macrolactin), ribosomally synthesized and post-translationally modified peptides (RiPPs) (e.g., lanthipeptides, lasso peptides, sactipeptides), polyketide-peptide hybrids (e.g., amicoumacin and ieodoglucomide), terpenes and alkaloids (Reimer and Bode, 2014; Li et al., 2015; Bartholomae et al., 2017; Greule et al., 2017; Kaspar et al., 2019; Hug et al., 2020). Nonetheless, the *Bacillus* spp. with broad-spectrum bacteriostatic activity utilized to control shrimp diseases especially AHPND are poorly documented and it is less clear by what mode of action these bacteria mitigate infections and facilitate shrimp culture.

Therefore, the present study aimed to explore the biologically active natural products produced by *Bacillus* spp. against AHPND and other vibriosis in Pacific white shrimp (*Litopenaeus vannamei*). To fulfill this goal, we chose a bacterial strain named BSXE-1601 as a therapeutic agent candidate. This strain was isolated from both the intestine of shrimp and the sediments of shrimp culture ponds during August 2013 at Lianyungang City, Jiangsu Province, China. In the present study, an assessment of the antagonistic activity *in vitro* was conducted to identify the therapeutic potential of the strain BSXE-1601 against vibriosis. Moreover, a bioactive metabolite responsible for this anti-*Vibrio* activity was isolated, structure analyzed, and bioactivity determined. To interrogate the potential biosynthetic pathways of the bioactive metabolites and the biosynthetic capability of the strain BSXE-1601, the whole genome sequencing of the strain BSXE-1601 was conducted. Our whole-genome analysis of BSXE-1601 provides a better understanding of the mechanisms involved in the anti-*Vibrio* properties and its high potential to be used as a multifunctional biological agent in aquaculture by suppressing pathogenic microbes of shrimp.

## Materials and Methods

### Bacterial Strain Identification

BSXE-1601 was obtained from the Microbial Culture Collection Center, Laboratory of Aquaculture Ecology, Ocean University of China (also preserved in China General Microbiological Culture Collection Center, CGMCC NO. 15949). The morphological, physiological, and biochemical characteristics of the strain BSXE-1601 were tested according to Bergey’s Manual of Systematic Bacteriology (Krieg and Holt, 1984). The genetic identification was performed using 16S rRNA gene sequencing as described earlier with some modifications (Gomez-Gil et al., 2012). Briefly, the DNA of the strain BSXE-1601 was extracted by simple boiling (Ribeiro et al., 2016). Then polymerase chain reaction (PCR) amplification of the 16S rRNA gene was undertaken using universal primers (27F, AGAGTTTGATCMTGGCTCAG; 1491R, TACGGYTACCTTGTTACGACTT) with the following amplification program: one cycle at 94°C for 2 min followed by 35 cycles at 94°C for 1 min, 55°C for 1 min 30 s, 72°C for 2 min and a final extension step at 72°C for 5 min. After visualizing the PCR products using 1% gel electrophoresis at a constant voltage of 110 V, fragments were sequenced by Sangon (Shanghai, China). Nucleotide sequence was subjected to homology search using the BLAST software of the NCBI. The phylogenetic tree was constructed by the neighbor-joining (NJ) method using MEGA 6.0 software according to Hall (2013).

### Safety Assessment of the Strain BSXE-1601

To identify the toxicity of the strain BSXE-1601 to shrimp, its safety in shrimp was identified in the laboratory via immersion, dietary, and injection administrations. The strain BSXE-1601 was applied at a concentration of 10^4^, 10^6^, and 10^8^ CFU ml^–1^ in the immersion assay, 10^4^, 10^6^, and 10^8^ CFU g^–1^ in the dietary assay, and 10^4^, 10^6^, and 10^8^ CFU shrimp^–1^ in the injection test. The detailed procedure is specified in “SI toxicity assay” in Supplementary Material.

### Aquatic Pathogenic Strains

The selected indicator bacteria were 10 common aquatic pathogenic strains (Table 1). *V. harveyi* SRTT9, *V. alginolyticus* AR-1, and *V. vulnificus* S01P2 were isolated from diseased *L. vannamei*. *V. splendidus* BSD11 and *Pseudoalteromonas* sp. LPE40 were isolated from diseased sea cucumber *Apostichopus japonicus*. These five strains were stored in the Microbial Culture Collection Center, Laboratory of Aquaculture Ecology, KLMME, Ocean University of China, Qingdao, China. *V. parahaemolyticus* 20130629002S01 isolated from the AHPND-infected *L. vannamei* were kindly provided by the Yellow Sea Fisheries Research Institute, Chinese Academy of Fishery Sciences, Qingdao, PR China. Moreover, *V. parahaemolyticus* 20130629002S01 was identified as an AHPND-causing strain (VP_*AHPND*_ 2S01 for short) (Dong et al., 2017a,b; Chen et al., 2018). *Edwardsiella tarda* HC01090721 and *Streptococcus iniae* NUF849 were kindly provided by the Laboratory of Pathology and Immunology of Aquatic Animals, KLMME, Ocean University of China, Qingdao, PR China and proved to be pathogenic strains for Japanese flounder *Paralichthys olivaceus* (Tang et al., 2010; Sheng et al., 2018). *Aeromonas hydrophila* AP40301 described as a pathogen of turbot *Scophthalmus maximus* (Xu et al., 2018) and *Shewanella marisflavi* AP629 considered as a novel pathogen of sea cucumber *A. japonicus* (Li et al., 2010) were both kindly provided by the Key Laboratory of Mariculture and Stock Enhancement in North China’s Sea, Agriculture Ministry, PRC, Dalian Ocean University, Dalian, China.

**TABLE 1:** Aquatic animal pathogenic strains used in this study.

**Pathogenic strain**	**Aquatic animal species (source of the strain)**	**References**
*Vibrio alginolyticus* AR-1	Pacific white shrimp (*Litopenaeus vannamei*)	–
*Vibrio harveyi* SRTT9		–
*Vibrio vulnificus* S01P2		–
*Vibrio parahaemolyticus* 20130629002S01		Dong et al., 2017a,b
*Vibrio splendidus* BSD11	Sea cucumber (*Apostichopus japonicas*)	–
*Pseudoalteromonas* sp. LPE40		–
*Shewanella marisflavi* AP629		Li et al., 2010
*Aeromonas hydrophila* AP40301	Turbot (*Scophthalmus maximus*)	Xu et al., 2018
*Edwardsiella tarda* HC01090721	Japanese flounder (*Paralichthys olivaceus*)	Tang et al., 2010
*Streptococcus iniae* NUF849		Sheng et al., 2018

*The references of the pathogenic strains isolated by our lab are not shown here.*

### Microbial Fermentation

A single colony of the strain BSXE-1601 grown on nutrient agar (NA) at 28°C overnight was inoculated into a 500 ml Erlenmeyer flask containing 100 ml of nutrient broth medium. After incubating at 28°C overnight in an aerobic atmosphere on a rotary shaker with 180 rpm, 1 ml of cultural liquid was transferred as a seed into 100 ml of nutrient broth (NB) medium. The flasks were incubated at 28°C with 180 rpm overnight. Similarly, all of the aquatic pathogenic strains used for bacteriostatic experiments were grown in LB_35_ medium at 28°C overnight under aerobic conditions.

### Assessment of Antagonistic Activity *in vitro* Based on Agar Diffusion Assay

The antagonistic activities of culture broth and step purification fractions were evaluated by an agar well diffusion method (Lertcanawanichakul and Sawangnop, 2008). The inoculum was prepared using the 10 selected pathogenic strains grown overnight in LB broth medium and adjusted to a concentration of 10^6^ CFU ml^–1^. Aliquots of 100 μl of the culture broths were inoculated onto LB nutrient agar medium. The wells (diameter, 6 mm) were then punched in each plate and a 100 μl aliquot of strain BSXE-1601 culture broth or step purification fractions was filled into each well. The plates were aerobically incubated at 28°C for 24–48 h. The inhibition zones were then measured. Well diffusion tests were performed in triplicate for each strain.

### Assessment of Antagonistic Activity *in vivo* Based on a Feeding Assay

#### Experimental Diets

To check the resistant activity of the strain BSXE-1601 against vibrios *in vivo* in shrimps, a feeding test was conducted in the lab.

Basal diet (control diet) was formulated with commercial shrimp food (No. 2 feed for *L. vannamei*, from Nantong Chia Tai Co., Ltd, Nantong, China), covered with a layer of sodium alginate and fish oil (3.15 g 500 g^–1^ and 4.2 ml 500 g^–1^, respectively) (Sun et al., 2012). Strain BSXE-1601 was cultured overnight in NB medium as described above, and then harvested by centrifugation (2800 × *g* for 10 min). The fresh cells were resuspended in sterile normal saline, and then sprayed to the surface of commercial shrimp food, and the mixture was covered with a layer of sodium alginate and fish oil (the same ratio with control diet). On basis of basal diet, two treatment diets were supplemented with 3.5 × 10^7^ and 3.5 × 10^8^ CFU g^–1^ of strain BSXE-1601 (named as BSXE07 and BSXE08). All the diets were kept at 4°C for using later.

#### Experimental Animals and Feeding Experiment

*L. vannamei*, obtained from hatchery of Baorong Aquatic Science and Technology Development Co., Ltd. (Qingdao, China), were fed with basal diet and acclimated simultaneously for 10 days by increasing salinity 2‰ per two days from 21 to 30‰. Then 108 similar-sized individuals (4.00 ± 0.07 g) which had been starved for 24 h were randomly distributed into 9 aquariums (53 cm × 28 cm × 34 cm, 50 l), with a density of 12 shrimps per aquarium. The shrimps were fed daily at 8 am and 5 pm for 7 days, with the feeding amount of 4–5% of shrimp weight. Each experimental group (BSXE07 and BSXE08) was repeated at three times. Uneaten feed and feces in the tank were collected by pipetting before the next feeding. During the feeding trial, the environmental conditions were suitable for shrimps (temperature, 23 ± 1°C; salinity, 30‰; pH, 8.0 ± 0.1; dissolved oxygen, >5 mg l^–1^). The food consumption and mortality were recorded daily.

#### Sample Collection

At day 4 and day 7, three shrimps were selected from each aquarium. Hepatopancreas from surface-disinfected shrimp were aseptically removed and homogenized. A series of 10-fold dilutions was prepared using sterile normal saline, and 0.1 ml from each dilution was plated on TCBS agar using spread plating. *Vibrio* species were enumerated after a 48-h-incubation at 28°C.

### Characterization of Molecules Responsible for the Antagonistic Activity

#### Preliminary Isolation

(i) Preparation of cell-free supernatant (CFS). The overnight culturing broth of the strain BSXE-1601 was centrifuged at 448 × *g* for 10 min. The supernatant was treated with filtration using a 0.22 μm Millipore filter to obtain CFS, and the bacteria were resuspended in normal saline. The inhibitory activities of both the CFS and cell resuspension solution were determined by the agar well diffusion method as described before, using the strain VP_*AHPND*_ 2S01 as the indicator bacterium.

(ii) Preliminary purification of CFS by ammonium sulfate precipitation. The strain BSXE-1601 CFS was subjected to ammonium sulfate precipitation to obtain protein precipitation and deproteinized supernatant. Protein precipitate was solubilized in 0.01 M PBS (pH 7.2) and then dialyzed to remove salt to obtain dialysate. The inhibitory activities of both the dialysate and deproteinized supernatant were determined by the agar well diffusion method, using the strain VP_*AHPND*_ 2S01 as the indicator bacterium, contrasted with untreated CFS.

#### Physicochemical and Enzymatic Treatments

For thermostability assay, the CFS sample from the strain BSXE-1601 was subjected to heat treatment for 15, 30, 45, and 60 min at 60, 80, and 100°C, respectively. For pH stability, the strain BSXE-1601 CFS was incubated for 6 h at 28°C at different pH values, adjusted with NaOH and HCl. Sensitivity to proteinase K was tested by incubation for 30, 60, and 120 min at 37°C. The proteinase K was used at a final concentration of 1 mg ml^–1^. The antagonistic activity was determined before and after each treatment by the agar well diffusion method, using the strain VP_*AHPND*_ 2S01 as the indicator bacterium.

### Isolation and Characterization

The cell-free supernatant from the strain BSXE-1601 (5 l) was extracted with ethyl acetate (2 × 5 l) to give a yellow oily residue (6.3 g) by evaporation of menstruum in vacuum, which was subjected to chromatography over a silica gel column eluting with CH_2_Cl_2_/MeOH mixtures of a stably growing polarity (100:0, 50:1, 30:1, 20:1, 10:1, 1:1, v/v) to afford six parts (F1, 820 mg; F2, 3.60 g; F3, 187 mg; F4, 225 mg; F5, 126 mg and F6, 367 mg). Bioassay results indicated that F5 and F6 (10:1 and 1:1 CH_2_Cl_2_/MeOH) had a potent antagonistic effect against the selected pathogens. The mixture of Fraction 5 (126 mg) and Fraction 6 (367 mg) was subjected to Sephadex LH-20 chromatography (20 mm × 800 mm) and further purified by HPLC over ODS (40% MeCN-H_2_O, 0.05% TFA, v/v) to give compounds **1** (*t*_*R*_ 8.37 min; 5 mg), **2** (*t*_*R*_ 8.84 min) and **3** (*t*_*R*_ 9.05 min). Due to the fast interconversion between **2** and **3** in methanol under room temperature, the structures of **2** and **3** were determined from the spectroscopic data of their mixture (3 mg), through careful comparison of their ESI-MS and NMR with previously reported data (Iron et al., 1982; Itoh et al., 1982).

### Determination of MICs

The minimal inhibitory concentrations (MICs) of the purified antagonistic compounds for the 10 selected aquatic pathogenic strains were determined by the broth microdilution method according to Wiegand with a few modifications (Wiegand et al., 2008). Briefly, broth dilution used LB_35_ growth medium containing geometrically increasing concentrations (a twofold dilution series) of the antimicrobial substances, which was inoculated with 10^6^ CFU ml^–1^ of pathogenic cells. The MICs were considered to be the lowest concentration of antagonistic substances that caused total inhibition of bacterial growth. MICs were also determined in triplicate for each strain.

### Genome Sequencing, Assembly, and Annotation

The genomic DNA of the strain BSXE-1601 was extracted using the TIANamp bacteria DNA kit (Tiangen, China), and then sequenced with a 10-kb SMRTbell^TM^ template library using Pacific Biosciences (PacBio) Sequel Single-Molecule Real-Time (SMRT) sequencing. Pre-assembly error correction was performed with the hierarchical genome assembly process (HGAP) of SMRT Analysis software using default parameters. Then all of the obtained data were assembled into a single circular chromosome without gaps. The genome circle was prepared using Circos ver. 0.64^1^. Bacterial genes were predicted by Glimmer 3.02 software (Delcher et al., 2007) and gene functions were annotated by BLASTP using NR, KEGG, GO, COG, Swiss-Prot, and CAZy databases. The rRNAs and tRNAs were identified using RNAmmer 1.2^2^ and tRNAscan-SE 1.3.1^3^, respectively. The strain BSXE-1601 genome was analyzed using AntiSMASH (Blin et al., 2019) to predict the gene clusters coding for secondary metabolites. The Comprehensive Antibiotic Resistance Database (CARD) (Jia et al., 2016) was used to identify the genes relating to antimicrobial resistance.

### Statistics

The *Vibrio* counts in hepatopancreas of shrimp were subjected to two-way repeated measures ANOVA, followed by Bonferroni post-test, with BSXE-1601 adding concentration and time as factors. Statistical analyses were performed using the Statistical Package for the Social Sciences (SPSS) version 25.0 using a significance level of 5%.

## Results

### Identification of the Strain BSXE-1601

The strain BSXE-1601 was identified as a Gram-positive and spore-producing bacterium by Gram-staining and spore staining. The fully developed single colony of the strain BSXE-1601 grown on NA at 28°C for 24 h was moist, round or irregular in shape, and opaque and off-white in color. Further, this strain showed positive results for catalase, V-P test, and nitrate reduction test (Supplementary Table 1). The minimal inhibitory concentration of NaCl was 7% (Supplementary Table 1). With these features, the strain BSXE-1601 matched the physiological and biochemical properties of *Bacillus subtilis* (Krieg and Holt, 1984).

**FIGURE 1:**
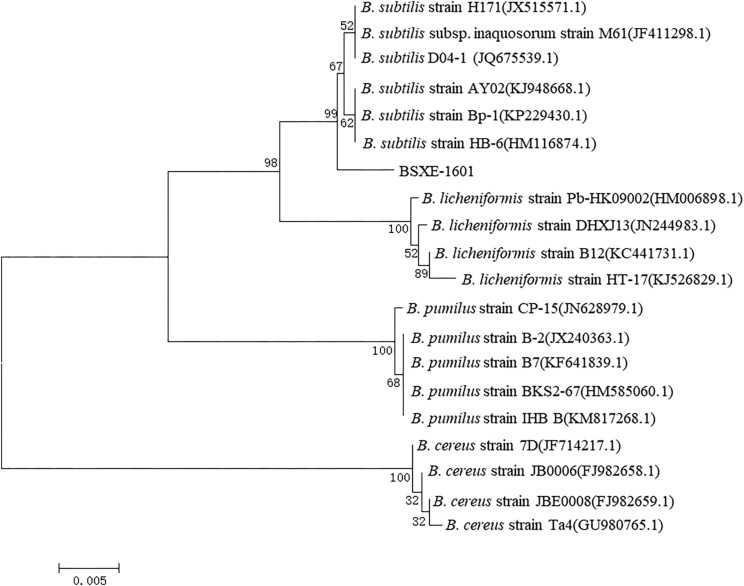
Phylogenetic tree of *Bacillus subtilis* BSXE-1601 and its closest relatives based on 16S rRNA sequence. The phylogenetic tree was constructed by the neighbor-joining (NJ) method using MEGA 6.0 software. The bootstrap values are shown at the branch points.

A 1,463 base pairs (bp) fragment of the 16S rRNA gene was amplified from the strain BSXE-1601. The BLAST matching analysis showed that the 16S rRNA gene sequence of the strain had a high similarity (99%) to that of *B. subtilis*. The phylogenetic tree was constructed by the neighbor-joining (NJ) method using MEGA 6.0 software, and it clearly showed that the strain BSXE-1601 clustered with members of *B. subtilis* strains (Figure 1).

### Safety Assessment of the Strain BSXE-1601

The safety assessment of the strain BSXE-1601 in shrimp was identified via the immersion, dietary, and injection administrations. None of these three detection methods caused diseases or mortality at the end of the experiment.

### *In vitro* Antagonistic Activities of the Strain BSXE-1601

According to the agar well diffusion method of antagonistic testing, of all the ten tested pathogenic strains, *B. subtilis* BSXE-1601 inhibited the activities of seven strains, including VP_*AHPND*_ 2S01, *V. alginolyticus* AR-1, *V. harveyi* SRTT9, *V. vulnificus* S01P2, *Pseudoalteromonas* sp. LPE40 and *S. marisflavi* AP629. The result shown in Table 2 indicated that BSXE-1601 certainly had potential antagonistic activity against aquatic pathogenic strains.

**TABLE 2:** The inhibition activity of *Bacillus subtilis* BSXE-1601 to different aquatic pathogens.

**Test organisms**	**The diameter of the inhibition zones (mm)**	**Antimicrobial susceptibility**
*V. vulnificus* S01P2	22.33	++
*V. harveyi* SRTT9	21.43	++
*V. alginolyticus* AR-1	19.99	++
*V. parahaemolyticus* 20130629002S01	19.03	++
*Pseudoalteromonas* sp. LPE40	11.08	+
*S. marisflavi* AP629	14.09	+
*V. splendidus* BSD11	<10	−
*A. hydrophila* AP40301	<10	−
*E. tarda* HC01090721	<10	−
*S. iniae* NUF849	<10	−

*“++”: susceptible; “+”: intermediate; “-”: non-susceptible or resistant.*

### *In vivo* Antagonistic Activities of the Strain BSXE-1601

**FIGURE 2:**
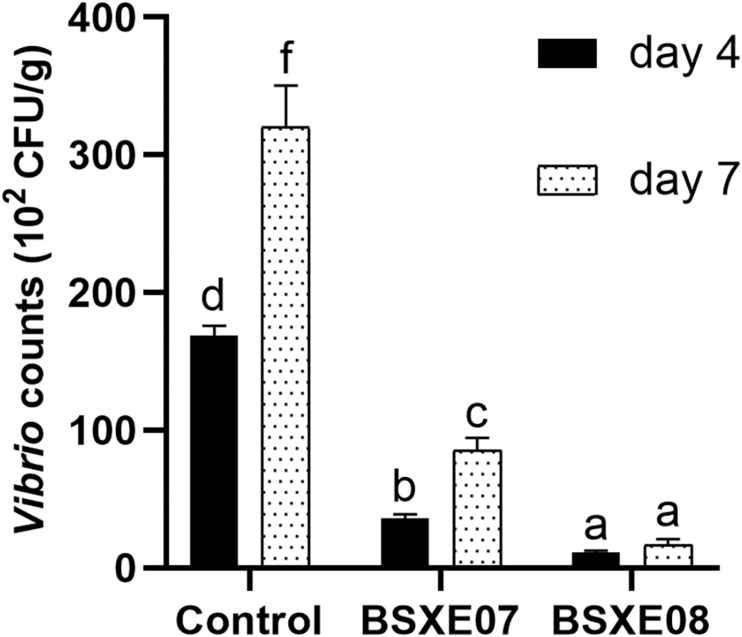
The resistant activity of BSXE-1601 against *Vibrio* spp. in the hepatopancreas of shrimp on day 4 and day 7 with different concentrations of BSXE-1601. Results are expressed as mean ± standard error of three replicates. Values marked with a different letter are significantly different (*P* < 0.05).

The resistant activities of BSXE-1601 against vibrios in the hepatopancreas of shrimp were checked on day 4 and day 7 with two concentrations of BSXE-1601 (3.5 × 10^7^ and 3.5 × 10^8^ CFU g^–1^). The results showed that both of the concentrations of BSXE-1601 (BSXE07 and BSXE08 treatments) had significant antagonistic effect on the vibrios in the hepatopancreas of shrimp as compared with the control at each time point (*P* < 0.05) (Figure 2). Comparing the vibrio counts on day 7 with that on day 4, there were no significant increase in BSXE08 but the significant increase in BSXE07 and the control group (*P* < 0.05), which demonstrated that the high concentration of the tested *B. subtilis* strain exhibited a promising antagonistic effect against vibrios.

### Antagonistic Compounds Extraction

#### Preliminary Identification of the Metabolites

The antagonistic testing of the preliminary purified metabolites showed that the inhibition zone of the cell-free supernatant (21.35 mm) was bigger than that of the resuspending solution (15.25 mm). Only deproteinized supernatant had the inhibition activity (19.54 mm) while the protein in the dialysate had no activity, which indicated that the inhibitory substances were extracellular and non-proteinaceous.

#### Effects of Heat, pH, and Proteinase K on Antagonistic Activity

The antagonistic activity against the strain VP_*AHPND*_ 2S01 was heat stable and remained unchanged after 60 min of incubation at 80°C. According to the inhibition zone, the antagonistic activity decreased slightly (14%) at 100°C after 60 min of incubation but kept active over a wide range of pH values, from 3 to 9. However, it was not resistant to strong alkaline pH, and the activity was noted to disappear at pH 12. Treatment with proteinase K did not affect the antimicrobial activity of the CFS.

### Structure Elucidation of the Compounds

**FIGURE 3:**
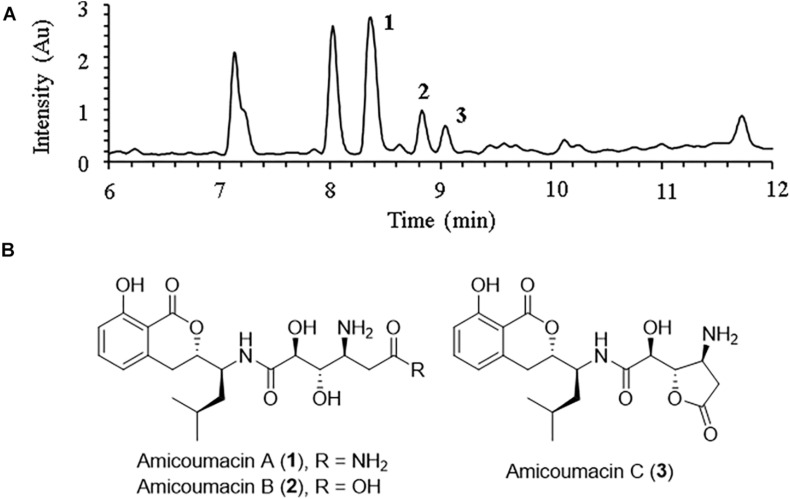
LC-MS profile of ethyl acetate (EA) extract from *B. subtilis* BSXE-1601 cell-free supernatant. **(A)** Amicoumacins A-C (**1**–**3**) were revealed from the chromatogram (DAD, λ = 314 nm) based on their specific m/z: 424 [M + H]^+^ for **1**, 425 [M + H]^+^ for **2** and 407 [M + H]^+^ for 3; **(B)** Structures of amicoumacins.

Careful analysis of the bioactive fractions obtained from *B. subtilis* BSXE-1601 using LC-MS revealed three isocoumarins like components from the chromatogram, which were further tested to be bioactive against several aquatic pathogens (Figure 3A). Large-scale fermentation, as well as chromatographic purification, allow further characterization of these three compounds as amicoumacins A-C (compounds **1**-**3**) (Figure 3B), based on a careful comparison of their 1D NMR and ESI-MS with the published data (Itoh et al., 1982; Table 3). We observed the fast interconversion between **2** and **3** in methanol under room temperature, which was consistent with the previous description (Itoh et al., 1982), partly due to the spontaneous intramolecular condensation between the -COOH and its γ-OH in **2**, as well as the hydrolysis of 5-membered lactone ring in **3**. The product ratio of **1**-**3** was calculated to be 4.8: 1.5: 1 based on their peak area.

**TABLE 3:** ^13^C NMR (150 MHz) data of amicoumacins A-C (**1**–**3**) in DMSO-*d*_6_.

**Pos.**	**1**	**2**	**3**
	**δ_*C*_, type**	**δ_*C*_, type**	**δ_*C*_, type**
1	169.0, C	169.0, C	169.1, C
2			
3	81.0, CH	80.8, CH	81.1, CH
4	38.6, CH_2_	38.7, CH_2_	38.7, CH_2_
5	118.6, CH	118.6, CH	118.6, CH
6	136.4, CH	136.5, CH	136.5, CH
7	115.3, CH	115.4, CH	115.5, CH
8	160.9, C	160.9, C	160.9, C
9	108.4, C	108.5, C	108.5, C
10	140.6, C	140.6, C	140.4 C
1′	21.5, CH_3_	21.5, CH_3_	21.6, CH_3_
2′	23.4, CH_3_	23.4, CH_3_	23.4, CH_3_
3′	25.3, CH	24.1, CH	24.1, CH
4′	31.5, CH_2_	29.2, CH_2_	29.2, CH_2_
5′	49.8, CH	47.7, CH	48.3, CH
6′			
7′	173.9, C	172.6, C	170.3, C
8′	70.9, CH	70.9, CH	72.0, CH
9′	73.8, CH	71.3, CH	84.0, CH
10′	51.2, CH	48.7, CH	48.6, CH
11′	33.3, CH_2_	32.3, CH_2_	34.2, CH_2_
12′	174.6, C	174.3, C	174.3, C

### MICs of Amicoumacins for Pathogenic Strains

Be consistent with the report of Iron et al. (1982), among the three amicoumacins, only amicoumacin A exhibited antibacterial activity (Table 4), and amicoumacins B and C were inactive against the pathogens (data didn’t show). The results revealed that amicoumacin A had good resistant activity against the tested bacterial pathogens of shrimp, especially VP_*AHPND*_ 2S01, *V. alginolyticus* AR-1, *V. harveyi* SRTT9, *V. vulnificus* S01P2, even at a low concentration of 1.25 μg ml^–1^.

**TABLE 4:** The minimal inhibitory concentrations (MICs) of amicoumacin A for pathogenic strains.

**Test organisms**	**M.I.C (μg ml^–1^)**
*V. vulnificus* S01P2	1.25
*V. harveyi* SRTT9	1.25
*V. alginolyticus* AR-1	1.25
*V. parahaemolyticus* 20130629002S01	1.25
*Pseudoalteromonas* sp. LPE40	1.25
*S. marisflavi* AP629	1.25
*S. iniae* NUF849	10
*A. hydrophila* AP40301	>10
*E. tarda* HC01090721	>10
*V. splendidus* BSD11	>10

### Genome Sequencing, Assembly, and Annotation

**FIGURE 4:**
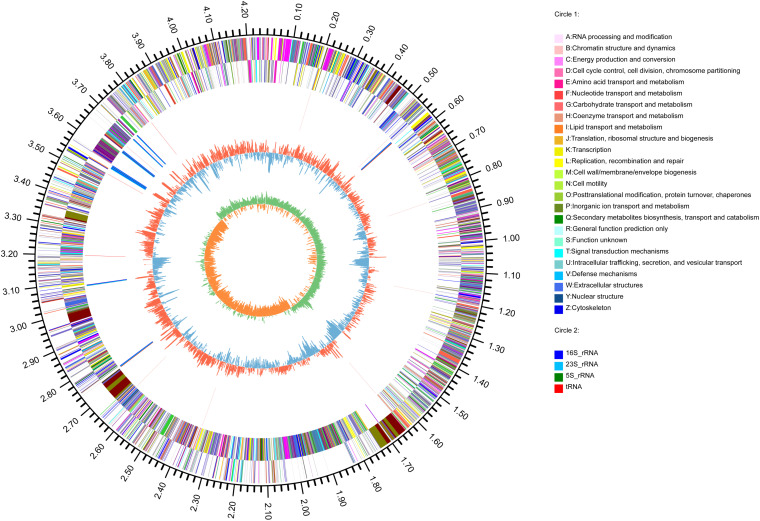
Circular representation of *B. subtilis* BSXE-1601 genome. The outer scale is in mega bases (Mb). Circle 1 (from outside to inside), the marker of genome size. Cycles 2 and 3, CDS with positive and negative chain, different colors represent different functional classifications; Circle 4, rRNA and tRNA; Circle 5, GC content, the higher value makes redder, the lower makes bluer. Circle 6, the GC-skew value, the algorithm is (G-C)/(G + C). Generally, when GC-skew > 0, the histogram is outward and expressed in red, GC-skew < 0, and the histogram is inward and expressed in blue.

The genome of *B. subtilis* strain BSXE-1601 consisted of a circular chromosome 4,242,126 bp in size with an average GC content of 44.03%. The further analysis predicted the chromosome contains 4,325 coding DNA sequences (CDSs), 30 rRNAs, and 86 tRNAs (Table 5 and Figure 4).

**TABLE 5:** General genome features of *B. subtilis* BSXE-1601.

**Features**	**Chromosome**
Genome topology	Circular
Assembly size (bp)	4,242,126
G + C content (%)	44.03
Protein coding sequences (CDS)	4,325
tRNA genes	86
rRNA genes	30
Secondary metabolite gene clusters	10
GenBank accession	CP028812

**FIGURE 5:**
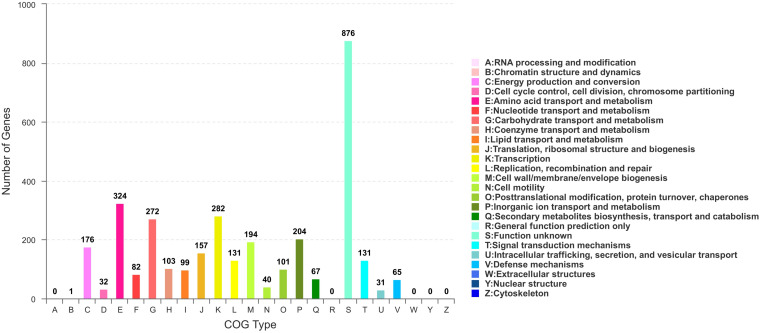
COG functional classification in *B. subtilis* BSXE-1601 coding sequences.

Based on COG analysis (Tatusov et al., 2003), 3315 proteins were classified into 21 functional categories. Among them, 1,327 proteins were associated with metabolism (40.03%): “amino acid transport and metabolism” (COG initial E, 9.77%), “carbohydrate transport and metabolism” (G, 8.21%), “inorganic ion transport and metabolism” (P, 6.15%), “energy production and conversion” (C, 5.31%), “coenzyme transport and metabolism” (H, 3.11%), “lipid transport and metabolism” (I, 2.99%), “nucleotide transport and metabolism” (F, 2.47%) and “secondary metabolites biosynthesis, transport, and catabolism” (Q, 2.02%) (Figure 5).

**FIGURE 6:**
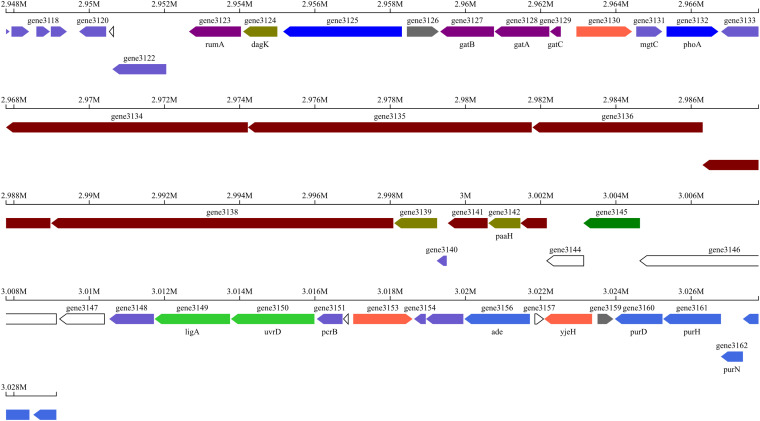
Specific gene structures in cluster 7.

10 putative gene clusters were identified to be responsible for secondary metabolite biosynthesis using antiSMASH v.5.0 (Table 6). Of these, cluster 7 displayed high similarity to the known amicoumacin biosynthetic gene cluster. Cluster 7 included 17 genes involved in the biosynthesis of amicoumacin A, which are 3 known genes mgtC, phoA, paaH and 14 unknown genes (Table 7). The gene structure of cluster 7 was shown in Figure 6. Moreover, the secondary metabolic gene cluster 1, cluster 4, cluster 8, cluster 9 and cluster 10 had high similarity to the biosynthetic gene clusters of bacillibactin, fengycin, surfactin, bacilysin and subtilosin A, respectively.

**TABLE 6:** Secondary metabolic gene clusters of *B. subtilis* BSXE-1601.

**Cluster**	**Type**	**Location**	**Similar cluster**	**Gene number**
1	NRPS	432092-481833	Bacillibactin biosynthetic gene cluster, NRPS	50
2	Type III PKS	1415819-1456916		48
3	Terpene	1506043-1527939		19
4	TransATPKS-NRPS	1625563-1757058	Fengycin biosynthetic gene cluster, hybrid	65
5	Terpene	2442478-2463282		25
6	TransATPKS-otherKS	2651018-2751176	Elansolid biosynthetic gene cluster, polyketide	42
7	Type I PKS-NRPS	2947790-3029128	Zwittermycin A biosynthetic gene cluster, hybrid	49
8	NRPS	3305557-3370946	Surfactin biosynthetic gene cluster, NRPS	48
9	NRPS	4064957-4106375	Bacilysin biosynthetic gene cluster, NRPS	48
10	Sactipeptide	4110050-4131660	Subtilosin A biosynthetic gene cluster, RiPP	21

**TABLE 7:** Secondary metabolic function genes related to the biosynthesis of amicoumacin A in *B. subtilis* BSXE-1601.

**Protein**	**Gene name**	**Size (aa)**	**Proposed function**	**Identity/similarity (%)**	**Accession No**
Orf 1	gene3147	397	MFS transporter	99.7	WP_003240131.1
AmiA	gene3146	1497	Non-ribosomal peptide synthetase	98.5	WP_003240128.1
AmiB	gene3145	501	Serine hydrolase	98.2	WP_003240126.1
AmiC	gene3144	327	Hypothetical protein	97.6	WP_003240124.1
AmiD	gene3143	233	Thioesterase	99.1	WP_003240123.1
AmiE^*b*^	gene3142 (paaH)	284	3-Hydroxybutyryl-CoA dehydrogenase	99.6	WP_003240121.1
AmiF^*b*^	gene3141	353	Hypothetical protein	100	WP_003240119.1
AmiG^*b*^	gene3140	90			WP_003240119.1
AmiH^*b*^	gene3139	380	Acyl-CoA dehydrogenase (NADP(+))	98.9	WP_003240115.1
AmiI	gene3138	3031	Hybrid non-ribosomal peptide synthetase/Type 1 polyketide synthase	98.9	WP_003240114.1
AmiJ	gene3137	889	Non-ribosomal peptide synthetase	99.2	WP_003240112.1
AmiK	gene3136	1508	Polyketide synthase	99.2	WP_003240111.1
AmiL	gene3135	2514	Polyketide synthase	98.8	WP_003240108.1
AmiM	gene3134	2142	Polyketide synthase	99.3	WP_003240106.1
AmiN	gene3133	333	Kinase	99.7	WP_003240104.1
AmiO	gene3132 (phoA)	459	Alkaline phosphatase	99.3	WP_003240102.1
Orf 2	gene3131 (mgtC)	230	Membrane component	99.6	WP_003240098.1

## Discussion

The growth of AHPND causative agent, VP_*AHPND*_ 2S01, as well as *V. alginolyticus* AR-1, *V. harveyi* SRTT9, *V. vulnificus* S01P2, *Pseudoalteromonas* sp. LPE40 and *S. marisflavi* AP629 was inhibited by the bioactive strain BSXE-1601 isolated from both shrimp intestine and shrimp culture ponds in this study. These bacterial pathogens are not only the most important causative agents of bacterial infections in shrimp but also responsible for fatal diseases in fish, shellfish and sea cucumber (Wang et al., 1999, 2000; Li et al., 2010, 2011; Austin et al., 2012; Petton et al., 2015; Zheng et al., 2016; Boonsri et al., 2017; Chandrakala and Priya, 2017; Karnjana et al., 2019; Nguyen et al., 2019). The isolated strain BSXE-1601 was then identified as *B. subtilis* by sequencing the 16S rRNA gene. Recently, *Bacillus* species have been frequently reported to have the antagonistic properties against aquatic pathogenic bacteria, such as *V. parahaemolyticus* (Vinoj et al., 2013; Xu et al., 2013), *V. harveyi* (Liu et al., 2014; Zokaeifar et al., 2014), *V. splendidus* (Zhao et al., 2012), *V. alginolyticus* (Zhang et al., 2011), *A. hydrophila* and *E. tarda* (Ran et al., 2012). Hence, *Bacillus* species presented a high interest as a promising therapeutic agent against vibriosis and other diseases in aquaculture (Aly et al., 2008; Ran et al., 2012; Hoseinifar et al., 2018).

The biologically active natural products are mainly responsible for the bacteriostatic properties of *Bacillus* spp. (Kaspar et al., 2019; Kuebutornye et al., 2019). In the past decade, a new complex of the isocoumarins group has been extracted from the cell-free supernatant of *Bacillus* spp. (Saeed, 2016). Amicoumacins were the most studied and well-characterized isocoumarins which are composed of the low molecular weight phenylpropanol derivative substances. Amicoumacin A was first discovered from *B. pumilus* by Itoh et al. (1981) and then from different *Bacillus* strains (Pinchuk et al., 2002; Zidour et al., 2017; Terekhov et al., 2018) and *Xenorhabdus bovienii* (Park et al., 2016). The strong anti-inflammatory and antiulcer properties of amicoumacin A were reported in the 1980s (Itoh et al., 1981; Iron et al., 1982), which was subsequently associated with its anticancer activity (Li et al., 2012; Prokhorova et al., 2016). Furthermore, amicoumacin A was documented to display a pronounced bactericidal activity against clinically relevant bacterial pathogens, such as *Helicobacter pylori* (Pinchuk et al., 2001) and the “superbug” methicillin-resistant *Staphylococcus aureus* (MRSA) (Berrue et al., 2009; Lama et al., 2012; Han et al., 2013; Terekhov et al., 2020). Noteworthily, besides the clinical applications, amicoumacin A produced by *B. pumilus* H2 also exhibited antagonistic effect toward the fish pathogen *V. vulnificus* CZ-A2 *in vitro* (Gao et al., 2017). The discovery gave a new insight into the mechanism of the anti-*Vibrio* activity of *Bacillus* spp. However, there was limited information about the mitigation infection of amicoumacin A on aquatic animal pathogens, and its activity against the representative strain VP_*AHPND*_ and other shrimp pathogens was not previously described.

In our research, the biologically active metabolite which was responsible for the antagonistic activity of *B. subtilis* BSXE-1601 was identified as amicoumacin A, according to its physicochemical characterization and NMR spectral data analysis. Furthermore, the MICs determination for familiar aquatic animal pathogens suggested that amicoumacin A was very effective on VP_*AHPND*_ 2S01, *V. alginolyticus* AR-1, *V. harveyi* SRTT9, *V. vulnificus* S01P2, *Pseudoalteromonas* sp. LPE40 and *S. marisflavi* AP629, which was matched by the antagonistic properties of the strain BSXE-1601 culture broth. Our discovery provided the first evidence that amicoumacin A was highly active against the especially important Gram-negative shrimp pathogenic bacteria. These pathogens are also responsible for fatal diseases in fish, shellfish and sea cucumber (Li et al., 2010; Austin et al., 2012; Nguyen et al., 2019). Hence, our work may facilitate the application of the *B. subtilis* strain BSXE-1601 as the effective therapeutic for detrimental diseases in aquaculture.

To elucidate the mechanism of the action of amicoumacin A, many functional studies were conducted. Lama et al. (2012) found that when amicoumacin A was exposed to MRSA, it reduced the activity of murein hydrolase, called autolysin, which was highly needed in the regulation of continued wall extension and cellular division (Higgins et al., 1970; Gilpin et al., 1972). The recent research demonstrated that the mode of action of penicillin-induced explosive lysis of *Streptococcus pneumonia*, a decades-long puzzle, was also because of autolysin disorder (Flores-Kim et al., 2019). Interestingly, with the assistance of confocal microscopy and scanning electron microscopy, Gao et al. (2017) found that amicoumacin A, secreted by a marine probiotic strain, can damage the surface structure of *V. vulnificus* cells, as reflected by the formation of cell cavities, the presence of membrane holes, and consequent cell lysis. As a further step, Polikanov et al. (2014) suggested that amicoumacin A, as a potent protein synthesis inhibitor, could interfere with translation by locking the mRNA in the mRNA-binding channel of the 30S subunit. It indicated that amicoumacin A could interfere with bacterial and eukaryotic translation, which could be the reason for its antibacterial, antifungal, anticancer, and anti-inflammatory properties (Itoh et al., 1981; Canedo et al., 1997; Li et al., 2012; Polikanov et al., 2014; Prokhorova et al., 2016). Noteworthy, according to proteomics and metabolomics, Terekhov et al. (2018) revealed a distinct mechanism of *Bacillus* self-resistance to amicoumacin A, based on a subtle equilibrium of its activation and deactivation by phosphatase AmiO and kinase AmiN, respectively. Understanding the target mechanism of amicoumacin A and the on/off mode of activity switching has the potential to find novel ways of developing therapeutic treatments against aquaculture diseases.

To interrogate the potential biosynthetic pathway of amicoumacin A, the whole genome sequencing of the strain BSXE-1601 was conducted in our study. Gene clusters related to the biosynthesis of secondary metabolites were predicted based on the analysis with antiSMASH. It revealed that the secondary metabolite biosynthesis gene cluster 7 in the strain BSXE-1601 encoded a hybrid modular polyketide synthase-non-ribosomal peptide synthetase (PKS-NRPS), and 17 genes (gene3131-3147) in this cluster were proposed to be responsible for amicoumacin A biosynthesis. Inspection of this genetic locus identified a PKS-NRPS hybrid protein encoded by gene3138, three PKS encoded by gene3134-3136, and two NRPSs encoded by gene3137 and gene3146. This was consistent with the results of Li et al. (2015), who succeeded in the heterologous expression of amicoumacin A biosynthesis genes *amiA-O* and provided unequivocal evidence that the *ami* locus encodes amicoumacin biosynthesis. Moreover, with the successful heterologous expression of the amicoumacin biosynthetic gene cluster and the ability to readily inactivate individual *ami* genes, they identified that inactive preamicoumacin precursors were first synthesized and then hydrolyzed by the asparagine-specific peptidase (encoded by *amiB*) into the active component amicoumacin A.

Additionally, in the present study, with the antiSMASH analysis, another 9 secondary metabolic gene clusters were found in *B. subtilis* BSXE-1601 genomes: three encoding NRPSs, two terpene synthases (TSs), one Type III PKS, one trans-Acyl transferase polyketide synthetase (TransATPKS)-otherKS, one TransATPKS-NRPS hybrid, one sactipeptide synthase. The diverse secondary metabolic pathways indicated the high biosynthetic capability of the strain BSXE-1601. Cluster 1, 4, 8, 9, and 10 were highly similar to the known clusters encoding bacillibactin, fengycin, surfactin, bacilysin, and subtilosin A, all of which have antimicrobial/antibacterial activities (Arguelles-Arias et al., 2009; Chen et al., 2009). For instance, bacilysin is one of the simplest non-ribosomal peptides produced by *B. subtilis*, with antagonistic properties against several bacteria and fungi (Kenig and Abraham, 1976). Surfactin, the most powerful biosurfactant known to date, is another NRP which inhibits the biofilm formation of *Escherichia coli*, *Salmonella enterica*, *Proteus mirabilis*, and *Staphylococcus aureus* (Mireles et al., 2001; Liu et al., 2019). Subtilosin A is one of the ribosomally synthesized and post-translationally modified peptides from *B. subtilis*, which displayed activity against gram-positive and gram-negative bacteria (Shelburne et al., 2007; Huang et al., 2009). A large number of beneficial genes BSXE-1601 possessing means that the strain BSXE-1601 may have a high potential to be used as a multifunctional biological agent in aquaculture by suppressing pathogenic microbes of aquatic animals. However, the related functional genes still need to be verified. Since the yields of secondary metabolites differ in different species and even under different culture conditions, the isolation and identification of the biologically active metabolites from *B. subtilis* BSXE-1601 still need to be investigated for a better understanding of BSXE-1601. Furthermore, because of ecologically adaptive changes, members of *B. subtilis* exhibit considerable genomic diversity (Earl et al., 2008). Hence, to testify if the biosynthetic capabilities of amicoumacin A, bacillibactin, fengycin, surfactin, bacilysin and subtilosin A are BSXE-1601 specific, a further comparative genome study should be performed between BSXE-1601 and other *B. subtilis* strains.

## Conclusion

In conclusion, the results may provide new insights into the possible mechanism of antagonistic activity of *B. subtilis* strain BSXE-1601 against the bacterial pathogens of shrimp. Amicoumacins A, B, and C belonging to isocoumarins, were purified from the cell-free supernatant from the strain BSXE-1601 but only amicoumacin A was demonstrated to be responsible for this anti-*Vibrio* activity. Our reports gave the first description in which the resistances of amicoumacin A against shrimp pathogens, including the AHPND-causing strain *V. parahaemolyticus* VP_*AHPND*_ 2S01. However, whether amicoumacin A specifically targets the primary virulence factor mediating AHPND etiology and mortality in shrimp or not still needs to be clarified. The complete genome sequence of BSXE-1601 aided to elucidate amicoumacin A biosynthetic gene cluster and its potential metabolic pathway. This work may further facilitate the heterologous expression of the natural product amicoumacin A biosynthetic pathway to increase the yield of amicoumacin A. The predicted diverse secondary metabolic pathways indicated the ability of BSXE-1601 to produce a variety of effective antibacterial products, and its high potential to be applied as a multifunctional biological agent in aquaculture by suppressing pathogenic microbes of aquatic animals. Sustainable aquaculture may be assured with the exploration of this modest microbicidal technique.

## Data Availability Statement

The datasets presented in this study can be found in online repositories. The names of the repository/repositories and accession number(s) can be found below: https://www.ncbi.nlm.nih.gov/genbank/, CP028812.

## Ethics Statement

All animal experiments were carried out in accordance with the U.K. Animals (Scientific Procedures) Act, 1986, and associated guidelines, EU Directive 2010/63/EU for animal experiments.

## Author Contributions

XT and WZ provided the experimental ideas and the design of this study. DW did the experiment and wrote the manuscript with assistance of JL, GZ, and KZ. WW, WJ, HL, and VK helped to analyze the data and revise the manuscript. SD helped to revise the manuscript. All authors contributed to the article and approved the submitted version.

## Conflict of Interest

The authors declare that the research was conducted in the absence of any commercial or financial relationships that could be construed as a potential conflict of interest.

## Footnotes

1 http://circos.ca/

2 http://www.cbs.dtu.dk/services/RNAmmer/

3 http://www.vcru.wisc.edu/simonlab/bioinformatics/programs/install/trnascan-se.htm
