# Supplementary material for: Mechanism of the Potential Therapeutic Candidate Bacillus subtilis BSXE-1601 Against Shrimp Pathogenic Vibrios and Multifunctional Metabolites Biosynthetic Capability of the Strain as Predicted by Genome Analysis
Source: Front Microbiol. 2020 Oct 26;11:581802. doi: 10.3389/fmicb.2020.581802 (PMC7649127; doi:10.3389/fmicb.2020.581802)
Supplement: Supplementary file 1 [file Table_1.DOCX]

Supplementary Material

# Supplementary Information Text

# SⅠ toxicity assay

To check the toxicity of the *Bacillus subtilis* strain BSXE-1601 to shrimp, its safety in shrimp was identified in the lab via immersion, dietary, and injection administrations.

Experimental Strain

The strain BSXE-1601 was cultured in nutrient broth (NB) medium at 28 ℃ overnight in an aerobic atmosphere on a rotary shaker with 180 rpm, and then harvested by centrifugation (2800 × g for 10 min). The fresh cells were resuspended in sterile normal saline for later use.

Experimental Diets

Basal diet (control diet) was formulated with commercial shrimp food (White shrimp No. 2 feed, from Nantong Zhengda Technology Feedstuff Co., Ltd.), covered with a layer of sodium alginate and fish oil (3.15 g·500 g^-1^ and 4.2 ml·500 g^-1^, respectively) (Sun et al., 2012). The bacterial suspension was sprayed to the surface of commercial shrimp food, and the mixture was covered with a layer of sodium alginate and fish oil (the same ratio with control diet). So on the basis of the basal diet, three treatment diets were supplemented with 10^4^, 10^6^, and 10^8^ CFU·g^-1^ of the strain BSXE-1601. All the diets were kept at 4 ℃ for later use.

Experimental Animals

*L. Vannamei*, obtained from hatchery of Baorong Aquatic Science and Technology Development Co., Ltd (Qingdao, China), were fed with the basal diet and acclimated simultaneously for 10 days by increasing salinity 2 ‰ per two days from 21 ‰ to 30 ‰. Then 300 similar-sized individuals (4.00 ± 0.07 g) which had been starved for 24 h were randomly distributed into 30 aquariums (53 × 28 × 34 cm, 50 L), with a density of 10 shrimp per aquarium.

Safety assessment of the strain BSXE-1601

A safety assessment of the strain BSXE-1601 was conducted via immersion, dietary, and injection administrations according to Fu et al. (2007) with some modifications. For the immersion assay, the bacterial suspension was daily added into the shrimp culture water to obtain the final concentration of 10^4^, 10^6^, and 10^8^ CFU·ml^-1^, respectively. For the oral feeding assay, shrimp were fed daily at 8 am and 5 pm with the diets supplemented with 10^4^, 10^6^, and 10^8^ CFU·g^-1^ of the strain BSXE-1601, and with the feeding amount of 4%-5% of shrimp weight. For the injection test, shrimp were injected with the strain BSXE-1601 at 10^4^, 10^6^, and 10^8^ CFU per shrimp. The shrimp fed with only the basal diet were used as control and both test and control groups comprised 10 shrimp each in triplicate. The shrimp used for immersion and injection tests were also fed daily at 8 am and 5 pm with the basal diet. Uneaten feed and feces in the tank were collected by pipetting before the next feeding. During the feeding trial, the environmental conditions were suitable for shrimp (temperature, 23 ± 1 ℃; salinity, 30 ‰; pH, 8.0 ± 0.1; dissolved oxygen, > 5 mg·l^-1^). The immersion and injection tests lasted for 7 days, while the oral feeding assay lasted for 14 days. The mortality was daily recorded.

There was no disease or shrimp death happened during the toxicity assay, which indicates that the strain BSXE-1601 appeared to be nontoxic to *L. Vannamei* under the present experimental conditions.

# References

Fu, Y. W., Hou, W. Y., Yeh, S. T., Li, C. H., and Chen, J. C. (2007). The immunostimulatory effects of hot-water extract of *Gelidium amansii* via immersion, injection and dietary administrations on white shrimp *Litopenaeus vannamei* and its resistance against *Vibrio alginolyticus*. *Fish and Shellfish Immunology*, *22*(6), 673–685. https://doi.org/10.1016/j.fsi.2006.08.014

Sun, Y., Liu, F., Song X., Mai, K., Li, Y., and Huang J. (2012). Effects of adding probiotics in the feed on non-specific immune gene expression and disease resistance of *Litopenaeus* *vannamei*. *Oceanologia et Limnologia Sinica/Hai Yang Yu Hu Chao, 43*(4), 845-851.

**Table S1** Physiological and biochemical properties of the strain BSXE-1601.

| **Properties** | **BSXE-1601** | **Properties** | **BSXE-1601** |
| --- | --- | --- | --- |
| Motility | + | Arabinose | + |
| Gram stain | + | Propionate | - |
| Spore stain | + | Starch hydrolysis | + |
| Catalase | + | Nitrate reduction test | + |
| V–P test | + | Salt resistance | <7% |
| Mannitol | + |  |  |

Note: +, positive; -, negative.
